# MMTV-NeuT/ATTAC mice: a new model for studying the stromal tumor microenvironment

**DOI:** 10.18632/oncotarget.24233

**Published:** 2018-01-15

**Authors:** Hongyan Yuan, Xiaoyi Wang, Jin Lu, Qiongsi Zhang, Irina Brandina, Ilya Alexandrov, Robert I. Glazer

**Affiliations:** ^1^ Department of Oncology, Lombardi Comprehensive Cancer Center, Georgetown University Medical Center, Washington, DC 20007, USA; ^2^ ActivSignal, LLC, Natick, MA 01760, USA

**Keywords:** fibrosis, mammary tumorigenesis, NeuT, adipose tissue, chemokines

## Abstract

One of the central challenges in cancer prevention is the identification of factors in the tumor microenvironment (TME) that increase susceptibility to tumorigenesis. One such factor is stromal fibrosis, a histopathologic negative prognostic criterion for invasive breast cancer. Since the stromal composition of the breast is largely adipose and fibroblast tissue, it is important to understand how alterations in these tissues affect cancer progression. To address this question, a novel transgenic animal model was developed by crossing MMTV-NeuT mice containing a constitutively active ErbB2 gene into the FAT-ATTAC (fat apoptosis through targeted activation of caspase 8) background, which expresses an inducible caspase 8 fusion protein targeted to mammary adipose tissue. Upon caspase 8 activation, lipoatrophy of the mammary gland results in stromal fibrosis and acceleration of mammary tumor development with an increase in tumor multiplicity. Fibrosis was accompanied by an increase in collagen deposition, α-smooth muscle actin and CD31 expression in the tumor stroma as well as an increase in PD-L1-positive tumor cells, and infiltration by regulatory T cells, myeloid-derived suppressor cells and tumor-associated macrophages. Gene expression and signal transduction profiling indicated upregulation of pathways associated with cytokine signaling, inflammation and proliferation. This model should be useful for evaluating new therapies that target desmoplasia in the TME associated with invasive cancer.

## INTRODUCTION

Over the past decade, it has become increasingly apparent that the cell-centric hallmarks of cancer originally proposed [1] are exceedingly more complex, and must now take into account the multi-faceted role and interplay of multiple cell types in the tumor microenvironment (TME) [2–5]. Although the TME is emerging as an important determinant of tumorigenesis and as an attractive target for therapy [6], understanding the specific cellular and molecular changes in the TME associated with breast cancer risk remains one of the overarching challenges for the prevention and treatment of this disease. The breast is composed of several stromal elements, among which adipose and fibrotic tissue comprise the largest components. Breast density increases with an increasing ratio of glandular and fibrous tissue to adipose tissue, making mammographic diagnosis more difficult and increasing the likelihood of developing breast cancer [7–10]. Since stromal fibrosis is a histopathologic criterion for predicting invasive breast cancer [11, 12], metastasis [13] and the development of precancerous lesions [14], the identification of coordinated signaling pathways between tumor and stromal cells would greatly aid our understanding of their roles in tumorigenesis. During transition from pre-invasive to invasive ductal breast carcinoma, the TME undergoes extensive changes due to secretion of inflammatory factors by tumor cells and cancer-associated fibroblasts and macrophages [15, 16]. Stromal fibroblasts secrete chemokines, such as Cxcl1, which promote angiogenesis, growth, invasion [17] and metastasis in breast cancer [17, 18] as well as reduced survival in colorectal, bladder and prostate cancers [19–21]. Cxcl1 and Ccl2 [22] facilitate immune tolerance by recruiting and activating regulatory T cells (Treg) and myeloid-derived suppressor cells (MDSC), which reduce activation of CD8^+^ cytotoxic effector T cells [23, 24]. This outcome suggests that therapy targeting, in part, chemokine receptor pathways including Cxcl1/Cxcr2, may be an effective approach for reducing or preventing the tumor-promoting effect of the inflammatory TME [25, 26].

Thus far, there is a paucity of relevant animal models for studying the relationship between mammary fibrosis and tumorigeneses. To develop a better understanding of this relationship, we developed a unique erbB2 oncogenic mouse model in which stromal fibrosis could be induced conditionally by the selective ablation of mammary adipose tissue. Here we report our initial observations of the impact of stromal fibrosis on erbB2-mediated mammary tumorigenesis.

## RESULTS

### Induction of stromal fibrosis and tumor development

**Figure 1:**
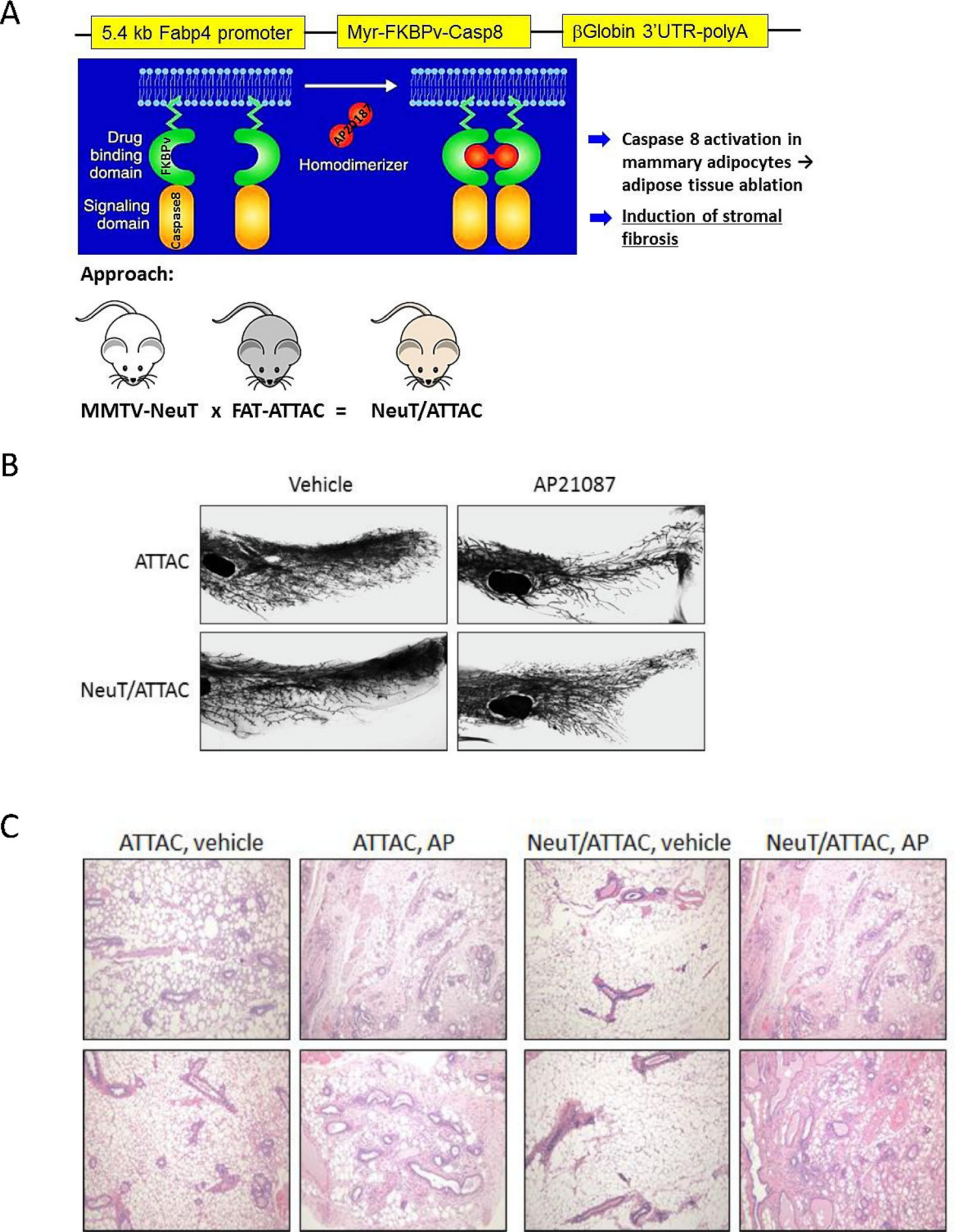
Conditional NeuT/ATTAC mice (**A**) Schematic of the generation of NeuT/ATTAC mice. FAT-ATTAC mice express a myristoylated-FKBPv-caspase 8 fusion protein under the control of the adipose-selective minimal Fabp4 promoter. Caspase is activated by dimerization of adjacent FKBPv domains (*green*) by the dimerizer AP21087 (*red*). Mouse color is for illustrative purposes and does not reflect genetic background. (**B**) Whole mounts of the mammary gland from FAT-ATTAC (*ATTAC*) or NeuT/ATTAC mice treated with vehicle or AP21087 for two weeks. Fat pad ablation increased ductal branching and reduced invasion of the fat pad. Magnification 10×, (**C**) H&E staining of the mammary gland treated with vehicle or AP21087 for two weeks as in (B). Two different tissue sections per group are shown. Loss of adipose tissue in ATTAC and NeuT/ATTAC mice following AP21087 treatment was accompanied by the initial stages of fibrosis. Treatment of MMTV-NeuT mice with AP21087 did not alter the composition of the mammary gland (not shown). Magnification 400×.

NeuT/ATTAC mice were generated as a conditional model of breast fibrosis, which is associated with high mammographic density and invasive breast cancer [7–9, 27]. In this model, desmoplasia is induced in the mammary fat pad of female mice by drug-induced dimerization of the FKBPv-caspase 8 fusion protein targeted to adipocytes by the dimerizer AP21087 [28] (Figure 1A). Whole mounts of the mammary gland from FAT-ATTAC or NeuT/ATTAC mice treated with vehicle or AP21087 three times per week for two weeks produced increased ductal branching and a reduction in fat pad invasion (Figure 1B). AP21087 treatment resulted in loss of mammary adipose tissue in FAT-ATTAC and NeuT/ATTAC mice, which was accompanied by the initial stages of fibrosis (Figure 1C). AP21087 treatment of mice lacking the FAT-ATTAC background did not alter the fat composition of the mammary gland or produce fibrosis (not shown).

**Figure 2:**
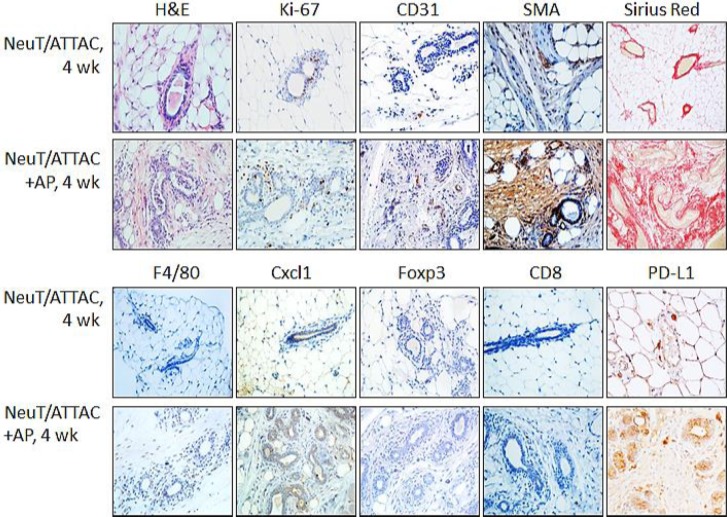
Induction of fibrosis in NeuT/ATTAC mice Mice at 6 weeks-of-age were injected i.p. with vehicle (*NeuT/ATTAC*) three times weekly for 5.5 months (*NeuT/ATTAC*) or with 0.4 mg/kg AP21087 *(NeuT/ATTAC+AP*) for 4 months. FFPE sections were stained with H&E, for collagen (PicroSirius Red) and with antibodies against Ki-67, CD31, α-smooth muscle actin (SMA), F4/80, Cxcl1, Foxp3, CD8 and PD-L1. Magnification 400×.

Longer term studies were then initiated to develop a baseline for the development of fibrosis. NeuT/ATTAC mice were treated with vehicle or AP-21087 three times per week for four weeks, which resulted in a pronounced loss of mammary fat and increased epithelial proliferation as determined by Ki-67 staining (Figure 2). These changes were accompanied by increased expression of biomarkers associated with angiogenesis (CD31) and fibrosis (SMA and collagen types I and III staining with PicroSirius Red). In addition, chemokine Cxcl1 and PD-L1 associated with immune tolerance were expressed in non-tumor tissue of the TME and correlated with the onset of macrophage and Treg infiltration as denoted by F4/80 and Foxp3 staining, respectively.

**Figure 3:**
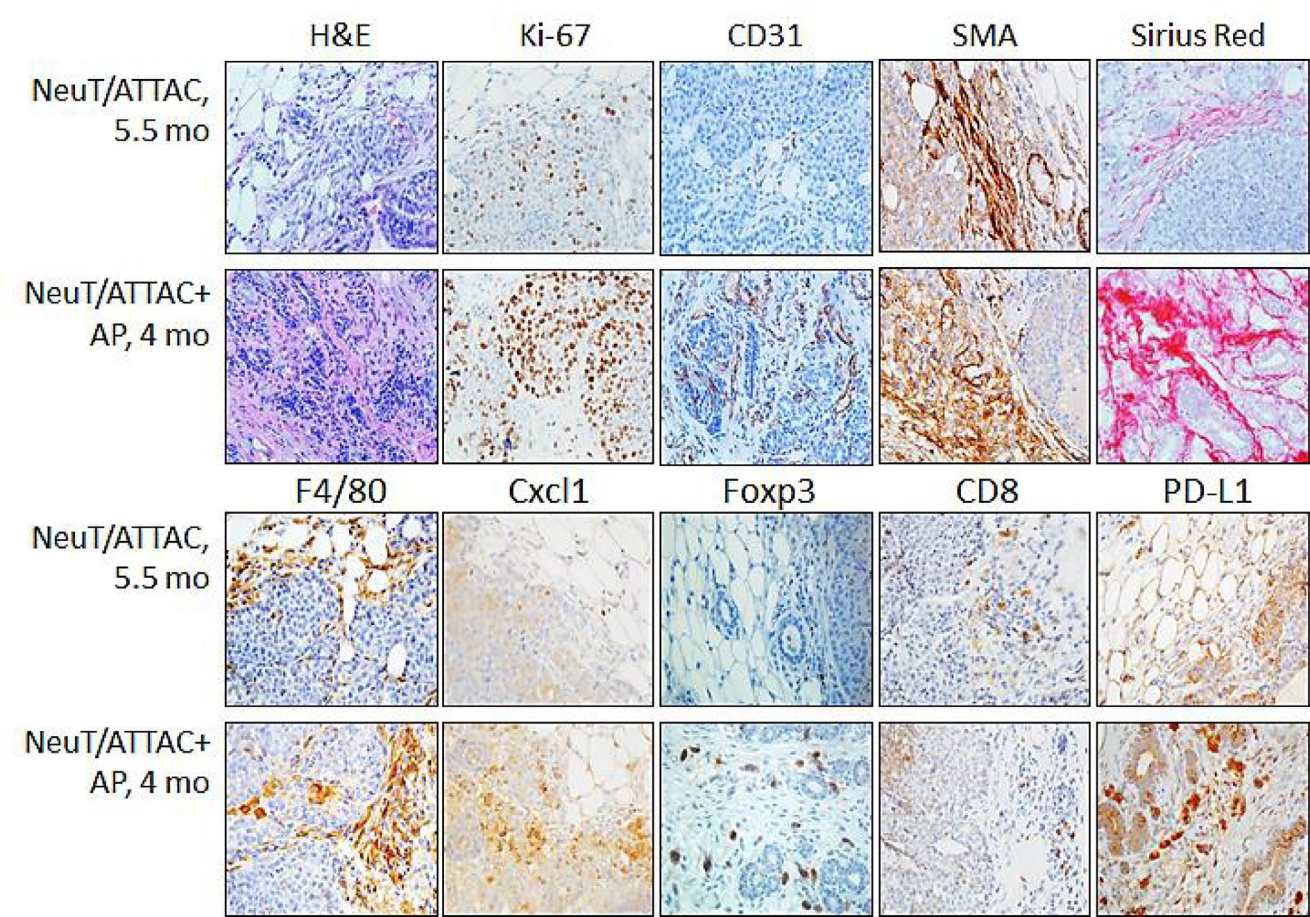
Induction of fibrosis in the mammary gland of NeuT/ATTAC mice following AP21087 treatment for four weeks Six-week-old NeuT/ATTAC mice were injected i.p. with vehicle (*NeuT/ATTAC*) or 0.4 mg/kg AP21087 *(NeuT/ATTAC+AP*) for four weeks. FFPE sections were stained as described in Figure 2. Magnification 400X.

**Figure 4:**
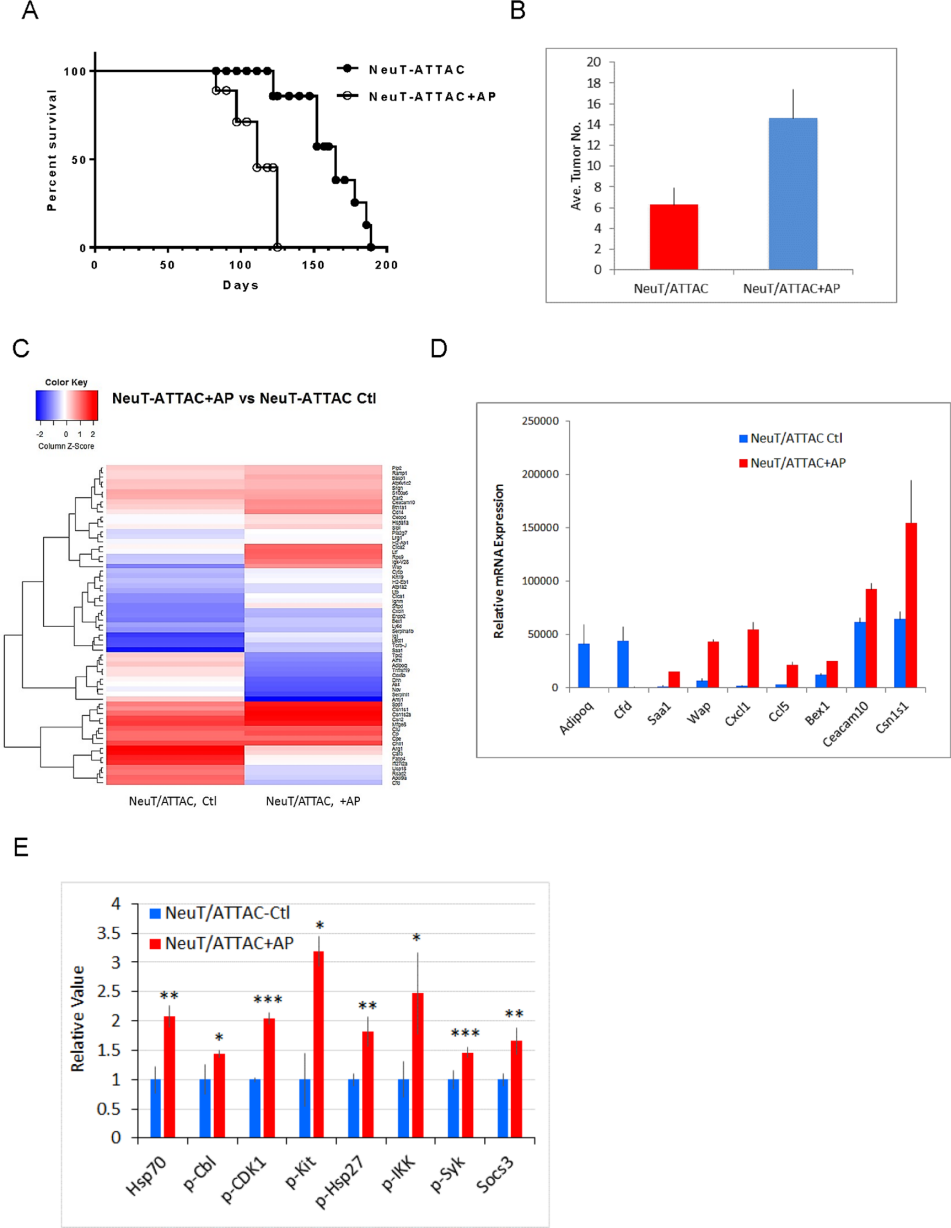
Survival and tumor multiplicity in NeuT/ATTAC mice after induction of fibrosis (**A**) NeuT/ATTAC mice were treated with AP21087 as described in Figure 2. Tumor latency was significantly reduced AP21087-treated NeuT/ATTAC mice (*N* = 9) vs. vehicle-treated mice NeuT/ATTAC mice (*N* = 10) using the Mantel-Cox log-rank test (*P <* 0.0001). (**B**) Tumor multiplicity was significantly increased from 6.3 ± 0.5 tumors/mouse (mean ± S.E.) in vehicle-treated NeuT/ATTAC mice to 14.6 ± 0.9 tumors/mouse in AP-21087-treated NeuT/ATTAC mice using the two-sided Student's *t* test (*P <* 0.001). (**C**) Heatmap of the changes in gene expression in NeuT/ATTAC mice treated with vehicle or AP21087 (Supplementary Table 2). RNA was prepared from mammary tumors from each of 5 mice per group and pooled for Affymetrix GeneChip analysis. (**D**) qRT-PCR analysis of genes selected from the Agilent array in Table 1. (D) qRT-PCR analysis of selected genes in Table 1 and Supplementary Table 2. (**E**) Immuno-paired antibody detection of signaling pathways in tumors from NeuT/ATTAC mice treated with AP21087 vs vehicle-treated mice as described in Figure 2. The epitopes recognized by the antibodies are described under Materials and Methods. ^*^P < 0.05, ^**^P < 0.02, ^***^P < 0.01.

To derive a better understanding between fibrosis and tumorigenesis, NeuT/ATTAC mice were treated with AP21087 or vehicle for four to six months in order to encompass the approximate median times of tumor formation (see Figure 4A). The longer treatment regimen resulted in more pronounced changes in the parameters measured after four weeks of AP21087 treatment, but resulted in greater Foxp3^+^ Treg and lesser CD8^+^ T cell filtration into the TME (Figure 3). Associated with the development of fibrosis was in a reduction in tumor latency from a median of approximately 160 days in mice treated with vehicle to 100 days following AP21087 treatment (Figure 4A), and an increase in tumor multiplicity from 7 tumors/mouse in vehicle-treated animals to 15 tumors/mouse in AP21087-treated animals (Figure 4B).

### Gene expression and cell signaling analysis

Gene expression analysis was next evaluated in mammary tumors developing at 4 months in AP21087-treated NeuT/ATTAC mice or at 5.5 months in vehicle-treated mice. Tumors from AP21087-treated NeuT/ATTAC mice resulted in marked changes in gene expression (Figure 3C), including upregulation of genes associated with adhesion (Clca1, Clca2, Krt19), inflammation/immunity (Saa1, CD14, Btn1a1, Ltb, Cxcl1, Ccl5, Saa1), invasion (Spp1), metabolism (Pla2g7), proliferation (Bex1, Basp1, Hsp1a1) and translation (Rps9), and a marked reduction in the adipose-specific genes Fabp4, Apol91 and Adipoq (Supplementary Table 2). Several of the changes in gene expression were confirmed by qRT-PCR (Figure 3D), and are summarized in Table 1. Similar gene expression profiling was conducted on mammary tissue after four weeks of AP21087 treatment (Supplementary Table 3). Interestingly, four weeks of AP21087 treatment produced a five-fold greater number of changes in gene expression as occurred in tumors after four months of treatment (Supplementary Table 3), including downregulation of 85% of metabolic genes, which accounted for more than 40% of the total changes in gene expression.

**Table 1:** Differentially expressed genes in tumors from NeuT/ATTAC mice 4 months after AP21087 treatment vs. tumors from control NeuT/ATTAC mice at 5.5 months

Gene Symbol	Gene Name	AP/Ctl	Function
**Adhesion/ECM**			
Clca1	chloride channel calcium activated 1	14.9	Adhesion, motility, interacts with integrinβ4
Clca2	chloride channel calcium activated 2	14.2	Adhesion, motility, interacts with integrinβ4
Krt19	keratin 19	4.7	Stem cell marker
Cpe	carboxypeptidase E	3.2	Adhesion, allograft rejection
**Inflammation/Immunity**			
Saa1	serum amyloid A 1	13.7	Acute phase protein, inflammation
Cd14	CD14 antigen	8.7	Macrophage/monocyte marker, TRAF/NFκB, inflammation
Btn1a1	butyrophilin, subfamily 1, member A1	6.0	Inhibits CD4 & CD8 T cell development
Ltb	lymphotoxin B	3.7	TRAF signaling, inflammation
Cxcl1	chemokine (C-X-C motif) ligand 1	3.6	Cxcr2 ligand, inflammation
Ccl5	chemokine (C-C motif) ligand 5	3.0	Ccr1/3/4/5 ligand, inflammation
**Invasion/Motility**			
Spp1	secreted phosphoprotein 1 (osteopontin)	11.8	Invasion, osteolysis
Slpi	secretory leukocyte peptidase inhibitor	3.6	Inhibits serine proteases
Enpp2	ectonucleotide pyrophosphatase/phosphodiesterase 2	3.2	Motility
S100a6	S100 calcium binding protein A6 (calcyclin)	3.0	Motility
**Metabolism**			
Pla2g7	phospholipase A2, group VII	3.7	Produced by inflammatory cells
**Proliferation**			
Bex1	brain expressed gene 1	4.6	Cell cycle progression
Basp1	brain abundant, membrane attached signal protein 1	4.3	Interacts with Hsp70, pre-mRNA processing, nuclear transport
Hspa1a	heat shock protein 1A (Hsp70)	3.7	Mitotic centrosome integrity, inhibits TGFβ signaling
Clu	clusterin	3.4	Inhibits apoptosis, degradation of IKKβ
Cd52	CD52 antigen	3.0	Inhibits tumor suppressor CDKN1B (p27)
**Signaling**			
Cytip	cytohesin 1 interacting protein	3.8	SOCS, Jak/Stat signaling
Ramp1	receptor (calcitonin) activity modifying protein 1	3.6	Prostaglandin E receptor 2, calcitonin & cAMP signaling
Lrg1	leucine-rich alpha-2-glycoprotein 1	3.2	Activates TGFβ signaling, angiogenesis, granulocyte differentiation
**Transcription/Translation**			
Rps9	ribosomal protein S9	32.5	40S ribosome-mRNA binding
Cebpd	CCAAT/enhancer binding protein (C/EBP), delta	4.3	Increases IL6 transcription and the inflammatory response
**Transport**			
Cp	ceruloplasmin	5.0	Iron transport
Plp2	proteolipid protein 2	3.3	Ion transport and chemokine binding.
Atp1a2	ATPase, Na+/K+ transporting, alpha 2 polypeptide	3.2	Na+/K+ transport, collagen polymerization

Shown are genes with ≥3-fold change in expression and a raw score ≥300 in either group as shown in Supplementary Table 2.

To obtain a better understanding of the changes in signal transduction occurring in tumors from NeuT/ATTAC mice after four months of AP21087 treatment, FFPE tumor sections were assessed for changes in 20 pathways by immuno-paired antibody detection (IPAD) for 73 phospho- and nonphospho-proteins (Figure 3E). IPAD analysis revealed significant upregulation in four major processes associated with proliferation (pCDK1), cytokine signaling (pCbl, pKit, pSyk and Socs3), protein processing (Hsp70/Hsp1a1, pHsp27/Hspb1) and inflammation (pIKK) in tumors from AP21087-treated mice versus vehicle-treated mice (Figure 3E).

## DISCUSSION

**Figure 5:**
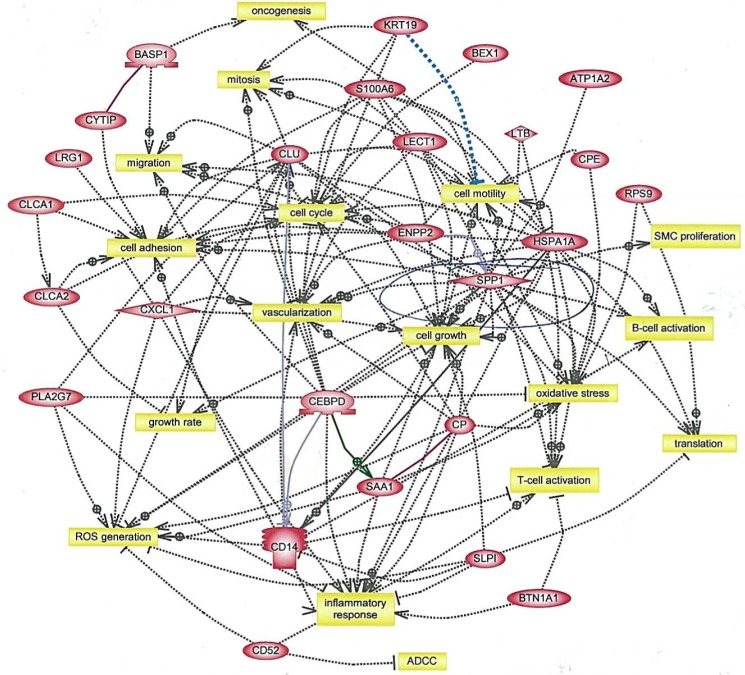
Interaction analysis of genes upregulated in tumors from AP21087-treated vs. vehicle-treated NeuT/ATTAC mice Mice were treated as described in Figure 2, and gene interactions were determined with Ariadne Pathway Studio version 9.1. The full list of gene expression changes in presented in Supplementary Table 2. ADCC, antibody-dependent cytotoxicity.

The present study describes a unique conditional model of mammary desmoplasia in which stromal fibrosis can be induced selectively in the mammary gland to study its link to tumor progression. It is well-established that the TME undergoes extensive changes during transition from pre-invasive to invasive ductal breast carcinoma, which results in changes in adaptive immunity [29] and poor outcomes [30]. The repertoire of stromal cell types, particularly cancer-associated fibroblasts, impacts every facet of transformation [5, 31–33], which was evident by interaction analysis of tumor genes upregulated by fibrosis (Figure 5). These changes encompassed every facet of tumor development, including adhesion, immune regulation, inflammation, mitosis, motility, oxidative stress and vascularization. Many of these changes were consistent with increased deposition of collagen associated with high breast density [27], the development of large fibrotic foci and early metastases in hormone receptor-negative breast cancer [11], identification of CD52, Spp1 and Cxcl14 in the stromal gene expression signature in breast cancer subjects [30], and the greater expression of Spp1 in HER2+ and triple-negative breast cancer [34].

One of the hallmarks of fibrosis is the aberrant secretion of chemokines which results in increased inflammation and activation of immune cells associated with immune tolerance [33]. These characteristics were indicative of tumors from NeuT/ATTAC mice, which exhibited increased Cxcl1 and Ccl5 expression (Table 1), but were not present in mammary tissue from mice treated for four weeks (Supplementary Table 3), suggesting that these changes are not early events accompanying fibrosis. This interpretation is supported by the lesser degree of immune cell infiltration at four weeks after AP21087 treatment (Figure 2) in comparison to longer treatment intervals (Figure 3). Cxcl1 and other chemokines [22] facilitate immune tolerance in part by recruiting and activating Treg and MDSC to block activation of CD8^+^ effector T cells [23, 24]. Cxcl1 modulates MDSC infiltration in prostate tumors [35] and rhabdomyosarcomas [26], is a prognostic marker of poor outcome in breast cancer [36] and promotes metastasis in an MDA-MB-231 breast cancer xenograft model [18]. Cxcr2 antagonists not only to reduce metastasis, tumor growth and improve response to chemotherapy in the xenograft model [18], but suppress inflammation-driven intestinal adenocarcinomas [25] and improve survival in a KRas-driven pancreatic ductal adenocarcinoma model [37]. Since many chemokine receptor antagonists have been developed and tested clinically in chronic inflammatory disorders [38, 39], it may be a useful strategy to employ these drugs as adjuvants for enhancing the efficacy of cancer therapy [25, 26].

IPAD analysis revealed that signaling mediated through Hsp70 and c-Kit were significantly upregulated in tumors from NeuT/ATTAC mice. These novel findings were not predicted by gene expression profiling and provide a unique technology for phenotyping tumors. In this regard, it is noted that the c-Kit inhibitor imatinib inhibited breast stromal fibroblasts and reduced tumor growth [40], and that c-Kit/CSF1R inhibitors target tumor-promoting M2 macrophages in the TME of solid tumors [41]. IPAD also identified heat-shock protein regulation as a potential therapeutic strategy as noted by the ability of the Hsp90 inhibitor geldanamycin to promote ErbB2 degradation in SKBR breast cancer cells [42].

Lastly, since loss of mammary fat elicited fibrosis, the reverse situation of inducing white adipose fat differentiation by PPARg agonists might be considered to suppress fibrosis [43]. However, mammary fibrosis in NeuT/ATTAC mice is distinct from adipose tissue fibrosis in the epididymal and mesenteric fat of male mice [43], where collagen VI predominates in contrast to collagen I and III in the mammary fat pad. Thus, it is uncertain if PPARγ agonists will have a beneficial effect as an anti-fibrotic therapy in breast cancer. An alternate approach to reduce mammary fibrosis and tumorigenesis in caveolin-1-deficient MMTV-PyMT utilized rapamycin since this model exhibits increased mTOR signaling [44]. Although this approach was also successful as an antitumor therapy in MMTV-PPARδ mice [45], NeuT/ATTAC mice do not display increased mTOR signaling or reduced caveolin-1 or -2 expression, and therefore rapamycin, at least in this context, would not be expected to have efficacy in NeuT/ATTAC mice.

In summary, induction of stromal fibrosis in the mammary gland accelerated tumor formation in MMTV-NeuT transgenic mice. This occurred in conjunction with upregulation of genes associated with growth, vascularization and immune regulation, and specifically with signaling pathways mediated through c-Kit, CDK1 and NFκB. These outcomes suggest several new therapeutic strategies to modify or reduce tumor progression.

## MATERIALS AND METHODS

### Animals

All animal studies were conducted under protocol 2016–1143 approved by the Georgetown University Animal Care and Use Committee in accordance with NIH guidelines for the ethical treatment of animals. MMTV-NeuT mice [46] were obtained from Jackson Labs (FVB-Tg(MMTV-Erbb2)NK1Mul/J), and exhibit 100% penetrance of mammary tumorigenesis by 6–7 months [47]. FAT-ATTAC mice on a C57BL/6 background were kindly provided by Dr. Philipp Scherer, University of Texas Southwestern [28, 48]. Mice were crossed into the FVB strain for four generations before crossing with MMTV-NeuT mice to produce NeuT/ATTAC mice. A schematic of the breeding is shown in Figure 1A.

### Induction of fibrosis

Animals at 6 weeks of age were treated weekly by i.p. injection of 0.4 mg/kg AP21087 dissolved in vehicle (4% ethanol, 10% PEG-400, and 1.75% Tween-20 in water) or with vehicle alone on Monday, Wednesday and Friday for the periods indicated. Mammary gland whole mounts were prepared to assess end-bud formation and ductal branching as previously described following two weeks of AP21087 treatment [45, 49, 50] (Figure 1B). Fat ablation was assessed by H&E staining of formalin-fixed paraffin-embedded (FFPE) sections that were prepared by the Histopathology & Tissue Shared Resource, Georgetown University.

### Histopathology and immunohistochemistry

Mammary tissue was excised and FFPE sections were prepared for antigen retrieval by incubation in 10 mM sodium citrate buffer (pH 6.0) for 20 min at a sub-boiling temperature in an electric steamer as previously described [45, 50, 51]. Endogenous peroxidase activity was quenched with 3% hydrogen peroxide for 10 min, and incubated for 30 min with blocking solution (10% goat serum in Tris-buffered saline), followed by incubation overnight at 4°C with the appropriate primary antibody diluted in blocking solution. Biotin-conjugated secondary antibodies were diluted in TBS containing 0.1% Tween-20 and incubated for 30 min at room temperature using the ABC Vectastain (Vector Laboratories) detection system and diaminobenzidine (Pierce), and slides were counterstained with Harris-modified hematoxylin (Thermo-Fisher, Inc.), dehydrated and mounted in Permount (Thermo-Fisher, Inc.). Antibodies and their dilutions for IHC are listed in Supplementary Table 1.

### Gene microarray analysis

At the appropriate intervals, animals were euthanized by CO_2_ asphyxiation, and tissue excised and snap-frozen in liquid nitrogen for gene expression profiling [45, 50–54]. Briefly, snap-frozen tissue was pulverized in a mortar and pestle and RNA extracted using an RNeasy Mini Kit (Qiagen) according to the manufacturer's protocol. RNA purity was assessed by the integrity of 18S and 28S rRNA using an Agilent microfluidic chip. Array analysis was carried out with cRNA prepared from equal amounts of RNA (1 μg) pooled from 3 mice per group. Biotin-labeled cRNA was fragmented at 94°C for 35 min and hybridized overnight to an Affymetrix mouse 430A 2.0 GeneChip^®^, and scanned with an Agilent Gene Array scanner. Affymetrix GeneChip^®^ Operating software 1.1 was used for grid alignment and raw data generation. A noise value (*Q*) based on the variance of low-intensity probe cells was used to calculate a minimum threshold for each GeneChip. Samples were averaged and data refined by eliminating genes with signal intensities <300 in both comparison groups, and heat maps were generated from ≥3-fold changes in gene expression normalized to control tissue using unsupervised hierarchical cluster analysis as previously described [55]. Gene expression data for mammary tissue after four months of AP21087 treatment are presented in Supplementary Table 2, and data for tumor tissue after four weeks of AP21087 treatment are presented in Supplementary Table 3. Gene interaction analysis of genes upregulated ≥3-fold following four months of AP21087 treatment was determined using Ariadne Pathway Studio version 9.1. Data sets have been deposited in the GEO public database under accession no. GSE78202.

### Quantitative real-time polymerase chain reaction (qRT-PCR)

Total RNA was extracted as described above and RNA (1 μg) from each of 3 samples per group was reverse transcribed using the Omniscript RT kit (Qiagen) as previously described [50–54]. PCR was performed in triplicate using an ABI-Prism 7700 (Applied Biosystems) with SYBRGreen I detection (Qiagen) according to the manufacturer's protocol. Amplification using the appropriate primers was confirmed by ethidium bromide staining of the PCR products on an agarose gel. The expression of each target gene was normalized to GAPDH and is presented as the ratio of the target gene to GADPH expression calculated using the formula, 2^−ΔCt^, where ΔCt = Ct^Target^-Ct^18s^ [53]. A list of primers used for qRT-PCR is presented in Supplementary Table 4.

### Immuno-paired antibody detection (IPAD) analysis

Triplicate 5 mμ sections of FFPE tissues were deparaffinized with xylene, rehydrated through a series of graded alcohol solutions and extracted for 10 min at 37°C with a buffer containing phosphate-buffered saline and 1% SDS using a proprietary procedure for IPAD analysis (https://www.activsignal.com/technology). The IPAD platform measures the activity of multiple signaling pathways in a single reaction in a 96-well high-throughput format. Approximately 150 ng of total protein lysate were analyzed per well to assess the activities of more than 70 target proteins in 20 signaling pathways using two distinct oligo-tagged antibodies per target. The combined tags were quantitated by digital PCR using a Fluidigm Biomark apparatus (Fluidigm). Phosphoantibodies detected pY700-Cbl, pY15-CDK1, pY730-c-Kit, pS78/82-HSPB1/Hsp27, pS176/180-IKK and pY525/526-Syk.

### Statistical analysis

Statistical significance of means±S.D. were evaluated using the two-tailed Student's *t* test at a significance of *P* < 0.05. Differences in tumor growth *in vivo* were determined by the unpaired two-tailed Student's *t* test at a significance of *P* < 0.05 using Prism GraphPad software.

## SUPPLEMENTARY MATERIALS TABLES






