# Correction: MMTV-NeuT/ATTAC mice: a new model for studying the stromal tumor microenvironment

**DOI:** 10.18632/oncotarget.26548

**Published:** 2018-12-28

**Authors:** Hongyan Yuan, Xiaoyi Wang, Jin Lu, Qiongsi Zhang, Irina Brandina, Ilya Alexandrov, Robert I. Glazer

**Affiliations:** ^1^ Department of Oncology, Lombardi Comprehensive Cancer Center, Georgetown University Medical Center, Washington, DC 20007, USA; ^2^ ActivSignal, LLC, Natick, MA 01760, USA

**This article has been corrected:** Due to a labeling error, the legends for Figures 2 and 3 were accidentally switched. The proper figure legends are given below. The authors declare that these corrections do not change the results or conclusions of this paper.

**Figure 2: Induction of fibrosis in the mammary gland of NeuT/ATTAC mice following AP21087 treatment for four weeks.** Six-week-old NeuT/ATTAC mice were injected i.p. with vehicle (*NeuT/ATTAC*) or 0.4 mg/kg AP21087 (*NeuT/ATTAC*+*AP*) for four weeks. FFPE sections were stained as described in Figure 2. Magnification 400×.

**Figure 3: Induction of fibrosis in NeuT/ATTAC mice.** Mice at 6 weeks-of-age were injected i.p. with vehicle (*NeuT/ATTAC*) three times weekly for 5.5 months (*NeuT/ATTAC*) or with 0.4 mg/kg AP21087 (*NeuT/ATTAC*+*AP*) for 4 months. FFPE sections were stained with H&E, for collagen (PicroSirius Red) and with antibodies against Ki-67, CD31, α-smooth muscle actin (SMA), F4/80, Cxcl1, Foxp3, CD8 and PD-L1. Magnification 400×.

Original article: Oncotarget. 2018; 9:8042-8053. https://doi.org/10.18632/oncotarget.24233

