# Supplementary material for: MMTV-NeuT/ATTAC mice: a new model for studying the stromal tumor microenvironment
Source: Oncotarget. 2018 Jan 15;9(8):8042–53. doi: 10.18632/oncotarget.24233 (PMC5814280; doi:10.18632/oncotarget.24233)
Supplement: Supplementary file 1 [file oncotarget-09-8042-s001.pdf]

MMTV-NeuT/ATTAC mice: a new model for studying the stromal tumor microenvironment

SUPPLEMENTARY MATERIALS

Supplementary Table 1: Antibodies used for IHC

Antibody	Catalog#	Source	IHC dilution
anti-PD-L1	17952-1-AP	Proteintech	200
anti-FAP	MBS176159	MyBiosource	100
anti-SMA	sc130617	Santa Cruz	100
anti-Ki67	CRM325	Biocare	50
anti-CD31	ab56299	abcam	100
anti-Cxcl1	sc-1374	Santa Cruz	120
anti-F4/80	14-4801-85	eBioscience	30
anti-CD8a	14-0808-82	eBioscience	40
anti-Foxp3	14-5773-82	eBioscience	50

Supplementary Table 2: Differentially expressed genes in tumors from NeuT/ATTAC mice following AP21087 treatment for 4 months vs. tumors from NeuT/ATTAC mice treated with vehicle for 5.5 months. See Supplementary_Table_2

Supplementary Table 3: Differentially expressed genes in the mammary gland of NeuT/ATTAC mice following AP21087 treatment for four weeks. See Supplementary_Table_3

Supplementary Table 4: List of primers for qRT-PCR analysis

Gene Accesion #	Primer Name	Sequence	Amplicon size (bp)
Adipoq	mAdipoq rRNA-F	TGAGACAGGAGATGTTGGAATG	110
NM_009605.5	mAdipoq rRNA-R	ACGCTGAGCGATACACATAAG	
Cfd	mCfd rRNA-F	TACGAGGACAAAGAAGTGGAAC	103
NM_013459.4	mCfd rRNA-R	CTCTGAGTTGATGCAGGACAT	
Clca1	mClca1 rRNA-F	GAGGTGTTCAGCAGCCATTA	101
NM_017474.2	mClca1 rRNA-R	GTCCCGTTACTCTGTCGATTAC	
Saa1	mSaa1 rRNA-F	GAGGACATGAGGACACCATTG	89
NM_009117.3	mSaa1 rRNA-R	CAGTCCAGGAGGTCTGTAGTAA	
Wap	mWap rRNA-F	CTTCTGCCCTTGGAATCTACTC	76
NM_011709.5	mWap rRNA-R	GTCGCTGGAGCATTCTATCTT	
Cxcl1	mCxcl1 rRNA-F	GTGTCTAGTTGGTAGGGCATAAT	94
NM_008176.3	mCxcl1 rRNA-R	CAGTCCTTTGAACGTCTCTGT	
Ccl5	mCcl5 rRNA-F	CCAGAGAAGAAGTGGGTTCAAG	100
NM_013653.3	mCcl5 rRNA-R	AGCAATGACAGGGAAGCTATAC	
Bex1	mBex1 rRNA-F	TCCAAAGATCAAGGCGTGAA	97
NM_009052.2	mBex1 rRNA-R	GCTCCCTTCTGATGGTATCTTG	
Ceacam10	mCeacam rRNA-F	CCTCAGCACATCTCCACAAA	82
NM_007675.4	mCeacam rRNA-R	GCAGGGCTCCAGTAAGTTAAA	
Csn1s1	mCsn1s1 rRNA-F	AGCCAGTCCTACTATCCACATA	99
NM_007784.3	mCsn1s1 rRNA-R	CAGTGCCTGATCCACTACAC	
GAPDH	mGAPDH rRNA-F	GGCAAATTCAACGGCACA	93
NM_001289726.1	mGAPDH rRNA-R	GTTAGTGGGGTCTCGCTCCTG	
